# Functionalized TiO_2_ nanoparticles labelled with ^225^Ac for targeted alpha radionuclide therapy

**DOI:** 10.1007/s11051-018-4181-y

**Published:** 2018-03-20

**Authors:** Edyta Cędrowska, Marek Pruszynski, Agnieszka Majkowska-Pilip, Sylwia Męczyńska-Wielgosz, Frank Bruchertseifer, Alfred Morgenstern, Aleksander Bilewicz

**Affiliations:** 1 Institute of Nuclear Chemistry and Technology, Dorodna 16, 03-195 Warsaw, Poland; 2 European Commission, Joint Research Centre, Department for Nuclear Safety and Security, 76125 Karlsruhe, Germany

**Keywords:** Targeted radionuclide therapy, ^225^Ac, Titanium dioxide nanoparticles, Substance P, Treatment cancer cells, Nanomedicine

## Abstract

The ^225^Ac radioisotope exhibits very attractive nuclear properties for application in radionuclide therapy. Unfortunately, the major challenge for radioconjugates labelled with ^225^Ac is that traditional chelating moieties are unable to sequester the radioactive daughters in the bioconjugate which is critical to minimize toxicity to healthy, non-targeted tissues. In the present work, we propose to apply TiO_2_ nanoparticles (NPs) as carrier for ^225^Ac and its decay products. The surface of TiO_2_ nanoparticles with 25 nm diameter was modified with Substance P (5-11), a peptide fragment which targets NK1 receptors on the glioma cells, through the silan-PEG-NHS linker. Nanoparticles functionalized with Substance P (5-11) were synthesized with high yield in a two-step procedure, and the products were characterized by transmission electron microscopy (TEM), dynamic light scattering (DLS) and thermogravimetric analysis (TGA). The obtained results show that one TiO_2_-bioconjugate nanoparticle contains in average 80 peptide molecules on its surface. The synthesized TiO_2_-PEG-SP(5-11) conjugates were labelled with ^225^Ac by ion-exchange reaction on hydroxyl (OH) functional groups on the TiO_2_ surface. The labelled bioconjugates almost quantitatively retain ^225^Ac in phosphate-buffered saline (PBS), physiological salt and cerebrospinal fluid (CSF) for up to 10 days. The leaching of ^221^Fr, a first decay daughter of ^225^Ac, in an amount of 30% was observed only in CSF after 10 days. The synthesized ^225^Ac-TiO_2_-PEG-SP(5-11) has shown high cytotoxic effect in vitro in T98G glioma cells; therefore, it is a promising new radioconjugate for targeted radionuclide therapy of brain tumours.

## Introduction

Despite of the predominant role of *β*^*−*^-particle emitters in radionuclide therapy trials (Iagaru et al. 2010), targeted radiotherapy based on *α*-particles is a promising alternative because they are highly cytotoxic agents, which deposit the whole of their energy within a few cell diameters (50–100 μm). Cell survival studies have shown that *α*-particle-induced killing is independent of oxygenation state (Wulbrand et al. 2013) or cell-cycle during irradiation and that as few as 1–3 tracks across the nucleus may result in cell death (Macklis et al. 1988; Sgouros et al. 2010). Additionally, the short range of *α*-particles corresponds to the dimensions of tumour micrometastases, allowing for localized irradiation of target cells with limited toxicity to surrounding healthy cells.

Recently, it has been found that in leukaemia, breast and brain cancer small subpopulations of tumour cells are able to self-renew and also to reconstitute the heterogeneous tumour-cell population (Al-Hajj et al. 2003; Singh et al. 2004). These, so called stem cancer cells, may be involved in the widespread metastatic dissemination of cancer (Wichal 2006). These findings have led to the suggestion that failure in tumour treatment may be associated with the failure to eradicate cancer stem cells (Al-Ejeh et al. 2011), especially that it has been found they are not sensitive to chemotherapy agents and to external and internal radiotherapy including *β*^*−*^-emitters (Al-Hajj et al. 2004). Given the properties outlined above, tumour stem cells are ideal targets for targeted *α*-particle therapy (TAT) (Sgouros and Song 2008).

There are hundreds of *α*-particle emitting radionuclides, but only a few have properties suitable for developing therapeutic radiopharmaceuticals: ^212^Bi (*t*_1/2_ = 60 min), ^213^Bi (*t*_1/2_ = 46 min), ^211^At (*t*_1/2_ = 7.2 h), ^212^Pb (*t*_1/2_ = 10.6 h), ^227^Th (*t*_1/2_ = 18.7 day), ^223^Ra (t_1/2_ = 11.4 day) and ^225^Ac (*t*_1/2_ = 9.9 day). However, most of the α-emitting radionuclides with optimal half-life for medical application, have one or more unstable daughter nuclides, which often emit *α*-particles as well. In principle such a cascade of *α*-particles can be very efficient in cancer treatment, but reliable delivery to the diseased site can be difficult to achieve. In the case of ^225^Ac, the most perspective radionuclide for TAT (Sattiraju et al. 2017; Zhu et al. 2017), the four consecutive decay daughters (i.e. ^221^Fr, ^217^At, ^213^Bi and ^209^Pb) receive recoil energies ranging from 105 to 160 keV as a direct result of the law of conservation of momentum. The recoil energy of firstly produced ^221^Fr is around 100 keV which is 1000 times greater than the energy of a chemical bond, implying that the recoiling daughter will always break free from the macrocyclic or acyclic chelating agents (Schwartz et al. 2011). Thus, produced daughter radionuclides are released from complexes, may accumulate in healthy organs and cause high toxicity which is a limiting factor in application of these radionuclides in targeted radiotherapy.

The new alternative strategy to sequester ^225^Ac and its daughters at targeted side is based on the use of nanocarriers. Such encapsulation is expected to prevent, or at least considerably reduce, the release of the recoiling daughters and by this to avoid undesired accumulation in healthy organs and tissues. Moreover, the use of nanocarriers may improve delivery of larger amounts of activity to the diseased site. There has been proposed several different types of nanostructures to retain ^225^Ac as well as its daughter radionuclides. Single and multi-layer liposomes have been evaluated; however, experimental studies revealed that their retention properties were poor with less than 10% of ^213^Bi retained (Sofou et al. 2004; Chang et al. 2008). Theoretical studies indicated that multi-walled polymerosomes could achieve around 80% of ^213^Bi retention, but their diameter has to be more than 800 nm (Thijssen et al. 2012). It would be very difficult for such large particles to extravasate/permeate/penetrate out of vasculature in vivo and also they can be clogged in microcapillary vessels. Woodward et al. (2011) developed 3–5 nm diameter monazite (LaPO_4_) nanoparticles as carriers for ^225^Ac, but these nanoparticles only partially retained daughters. More than ~ 50% of the ^221^Fr and ^213^Bi (from the decay of ^221^Fr) were released from the nanoparticle lattice. Addition of two layers of LaPO_4_ reduced leaching of ^221^Fr to 20% (Rojas et al. 2015). Similar nanocarrier based on lanthanide phosphate nanoparticles additionally coated with gadolinium and gold layers was developed by McLaughlin et al. (2013). Although multilayered nanoparticles exhibited higher retention properties, their synthesis becomes a multi-step and time-consuming procedure.

In the present work, for sequestration of ^225^Ac and its decay products, we propose to use titanium dioxide (TiO_2_) nanoparticles (NPs), which are inorganic cation exchangers exhibiting high affinity for ^225^Ac^3+^ and also ^211^Fr^+^ and ^213^Bi^3+^ cations. TiO_2_ NPs were conjugated with a fragment of substance P (5-11) for targeting NK1 receptors overexpressed in brain carcinomas. We examined the ability of TiO_2_ NPs to retain daughter products of ^225^Ac and investigated the cytotoxicity of the radiobioconjugate on human *glioblastoma* multiforme *cells* (T98G cells*)*.

## Materials and methods

### Reagents, radionuclide and cell line

The following chemical reagents were used directly without further purification: TiO_2_ nanoparticles (anatase, ~ 25 nm, Sigma Aldrich), ethanol (96%, POCH), acetic acid (POCH), phosphate-buffered saline (PBS, Amresco), sodium chloride (POCH), anhydrous dimethylformamide (DMF) and triethylamine (TEA) of 99% purity were from Sigma Aldrich, substance P (5-11) (Bachem), human serum (Sigma Aldrich, stored at − 20 °C), methoxyl silane and *N*-hydroxysuccinimide functionalized polyethylene glycol (silane-PEG- NHS, 2000 kDa, Nanocs) and deionized water (Millipore). ^225^Ac was produced by radiochemical separation from ^229^Th source as described before (Apostolidis et al. 2005; Zielinska et al. 2007). The activity of ^225^Ac was quantified using a high-resolution *γ*-spectrometry when it was in secular equilibrium with its daughters, typically next day after sample collection.

T98G cells (human glioblastoma multiforme cells; CRL-1690; American Type Culture Collection) were cultured in DMEM/F12 K medium (Gibco) enriched with 10% foetal calf serum (FCS, Biological Industries, Israel) and streptomycin (100 μg/mL) and penicillin (100 IU/mL) (Sigma Aldrich). Cells were grown in humidified atmosphere with 5% CO_2_ at 37 °C. Prior to their in vitro use cells was detached using trypsin-EDTA (0.25%) (Biological Industries, Israel).

### Instrumentation

The shape and diameter of titanium dioxide nanoparticles was determined by scanning electron microscopy (SEM, Zeiss) and transmission electron microscopy (TEM, LEO 912AB). The hydrodynamic diameter and zeta potential (*ζ*) were measured by dynamic light scattering (DLS, Malvern). The presence of surface coating of nanoparticles by PEG and substance P was determined by thermogravimetric analysis (TGA, Q500, TA Instruments). About 5 mg of dried powder was placed in a TGA furnace and the analysis was conducted from room temperature to 900 °C at a heating rate of 10 °C per min in the presence of inert (nitrogen) atmosphere.

Gamma-ray spectra of radionuclides were measured using coaxial HPGe detector (GX 1080) for gamma spectroscopy with multichannel analyzer DSA-1000 (Canberra Packard) with Genie 2000 software; detection range 10–5000 keV. High-performance liquid chromatography (HPLC) was performed using the ELITE LaChrom (VWR-Hitachi) system with L-2310 pump coupled to L-2455 diode array detector, radiometric (GabiStar, Raytest) detector and L-2350 column oven. Hamilton column (10 μm, 10 × 250 mm) eluted at the flow rate of 2 mL/min was used for the analytical and semi-preparative chromatography. The gradient elution system consisted of deionized water (A) and acetonitrile (B), both solvents contained 0.1% (*v*/*v*) trifluoroacetic acid (TFA). The gradient conditions were as follows: 0 to 70% solvent B in 0–15 min, 70 to 95% solvent B in 15–20 min.

### Conjugation of substance P (5-11) with PEG molecules

An equal molar amount of substance P (5-11) (SP(5-11)) was added to methoxyl silane and *N*-hydroxysuccinimide functionalized polyethylene glycol (silane-PEG-NHS) and dissolved in 250–300 μL DMF followed by 3 μL of TEA addition. Mixture was stirred for 72 h in an inert (nitrogen) gas atmosphere. After completion of the reaction (checked by HPLC), the solvent was removed under vacuum and the product (silan-PEG-SP(5-11)) was separated from unreacted substrates using above described HPLC system and conditions, and finally lyophilized.

### Functionalization of TiO_2_ surface with silane-PEG-SP(5-11)

The TiO_2_ NPs were functionalized with earlier synthesized conjugate silane-PEG-SP(5-11) according to the procedure described in literature (Hermanson 2008). The acetic acid was added to 50 mL of ethanol (96%) to adjust pH to 4.5–5.5. Then about 5 mg of silan-PEG-SP(5-11) conjugate was dissolved and solution was stirred for 5 min. Next, 10 mg of TiO_2_ NPs was added and the mixture was sonicated for 10 min followed by 2 h stirring at room temperature (RT). At the end, obtained product was washed several times with ethanol to remove unreacted silane-PEG-SP(5-11) and dried at 100 °C for 30 min.

### Labelling of TiO_2_-silane-PEG-SP(5-11) with ^225^Ac

^225^Ac^3+^ cations are adsorbed on the surface of TiO_2_ NPs through ion-exchange reaction on hydroxyl groups. For a typical labelling, ~ 1 mg of nanoparticles either bare TiO_2_ NPs or TiO_2_-silane-PEG-SP(5-11) NPs was suspended in 2 mL of ^225^AcCl_3_ (~ 100 kBq). The mixture was sonicated in an ultrasonic bath for 10 min and gently shaken on a circular stirrer for 1 h. After that time labelled NPs were centrifuged at 13000 rpm for 10 min, separated from supernatant and washed several times with deionized water. Finally, the product was suspended in 1 mL phosphate buffer (PB) and its activity measured on a γ-spectrometer to determine the labelling yield.

### Stability studies of labelled TiO_2_-silane-PEG-SP(5-11) NPs

In order to study leaching of ^225^Ac and its decay daughters, a portion of labelled TiO_2_-silane-PEG-SP(5-11) NPs was dialyzed against 20 mL of 0.1 M PBS or saline (0.9% NaCl) for 10 days. Every day a 1-mL aliquot was withdrawn and the percentage of liberated activity from each daughter radionuclide was determined by its characteristic γ-ray peak. The stability study of radiolabelled nanoparticles in cerebrospinal fluid (CSF) was conducted using a centrifugal method. A 10 μL sample of labelled TiO_2_-silane-PEG-SP(5-11) NPs was added to 200 μL of CSF, vortexed and incubated at 37 °C for 10 days. Every day, the probes with added nanoparticles were centrifuged and 100 μL of CSF was tested for activity of ^225^Ac and its decay daughters.

### Cytotoxicity evaluation

The impact of TiO_2_ nanoparticles conjugated with substance P(5-11) fragment and labelled with ^225^Ac on metabolic activity and proliferation of T98G cells was measured with 3-(4,5-dimetyl-2-thiazolyl)-2,5-diphenyl-2H-tetrazolium bromide (MTT, Sigma Aldrich) assay. The assay was performed as described by Lankoff et al. (2012). In brief, 1 day before the experiment, T98G cells were seeded in 96-well plates (TPP) at a density of 10^3^cells/well in 100 μL of culture medium. Cells were treated for 48, 72 and 96 h with increasing concentrations of non-radiolabelled TiO_2_ nanoparticles (0.0625–1 mg/mL) or the nanoparticles labelled with ^225^Ac (1.25–20 kBq/mL). At least three independent experiments in six replicate wells were conducted. After the described treatment, cell culture medium was removed and 100 μL of 3 mg/mL MTT solution was added to each well. After 3 h incubation at 37 °C the MTT solution was removed. Remaining insoluble formazan crystals were dissolved in 100 μL dimethylsulfoxide (DMSO, Sigma Aldrich) and absorbance of the solution was measured at 570 nm in plate reader spectrophotometer Infinite M200 (Tecan).

### Statistical analysis

At least three independent experiments in six replicate wells were conducted for each toxicity point. Difference between samples and control were evaluated using GraphPad Prism 5.0 software (GraphPad Software Inc., USA). Toxicological data were evaluated by Kruskal-Wallis One Way Analysis of Variance on Ranks (ANOVA) followed by post hoc Dunnet’s method. Differences were considered statistically significant when the *p* value was less than < 0.05.

## Results and discussion

Currently ^225^Ac is the most perspective radionuclide for targeted α-therapy in nuclear medicine. However, one significant factor limiting its use is the difficulty in sequestration of its decay products ^221^Fr and ^213^Bi at the targeted site. In our approach, we propose to use TiO_2_ nanoparticles as carriers for ^225^Ac and its decay daughters. Previous studies onTiO_2_ NPs properties indicated their high sorption affinity for 3+ metal cations (Metwally and Rizk 2014) and heavy alkali metal cations (Filipowicz et al. 2014) and facile functionalization and modification of their surface through hydroxyl groups (Bogdan et al. 2017). Therefore, these reports provided a compelling rationale to evaluate TiO_2_ NPs as potential carriers for stable retention of ^225^Ac and its decay daughters.

**Fig. 1:**
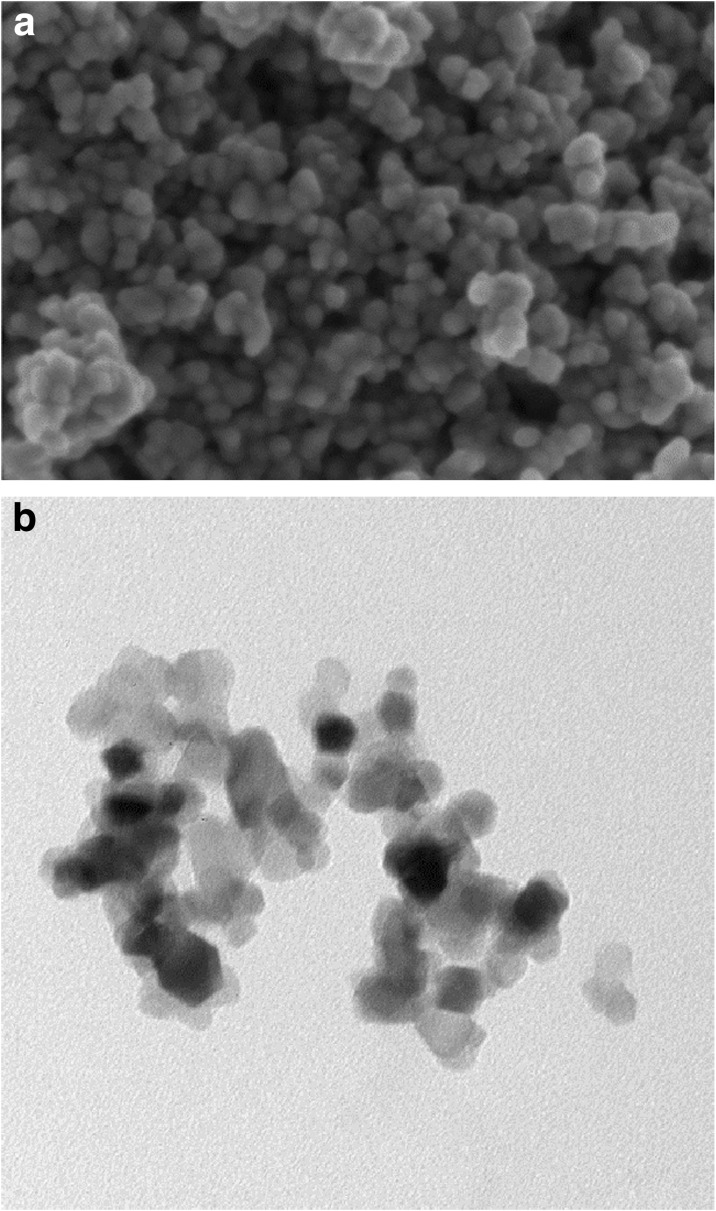
SEM (**a**) and TEM (**b**) images of bare TiO_2_ nanoparticles

The morphology, shape and diameter of bare TiO_2_ NPs were characterized by SEM and TEM microscopy. The TiO_2_ NPs exhibit nearly spherical morphology and their diameter ranges from 12 to 25 nm, as depicted on Fig. 1.

In order to synthesize a potential radiopharmaceutical exhibiting affinity to NK1 receptors overexpressed on glioma cells, attachment of an active biomolecule is required, in our case, an undecapeptide regulatory neuropeptide, substance P (SP). However, the native SP is metabolized in vivo due to enzymatic cleavages. The fragment SP(5-11) is the most abundant metabolite of native SP and it is still biologically active as the binding site with NK1 receptors is localized in the region of 7–11 amino acids. We have previously shown that this fragment radiolabelled with different radionuclides exhibit high affinity (IC_50_ = 38 nM) and interaction with NK1 receptors on T98G cells (Piotrowska et al. 2017; Lyczko et al. 2017). Additionally, fragment SP(5-11) contains l-glutamine in position 5 what allows coupling of silane-PEG-NHS via NHS-active ester to form bioconjugate that further is used to functionalize the TiO_2_ NPs surface.

**Fig. 2:**
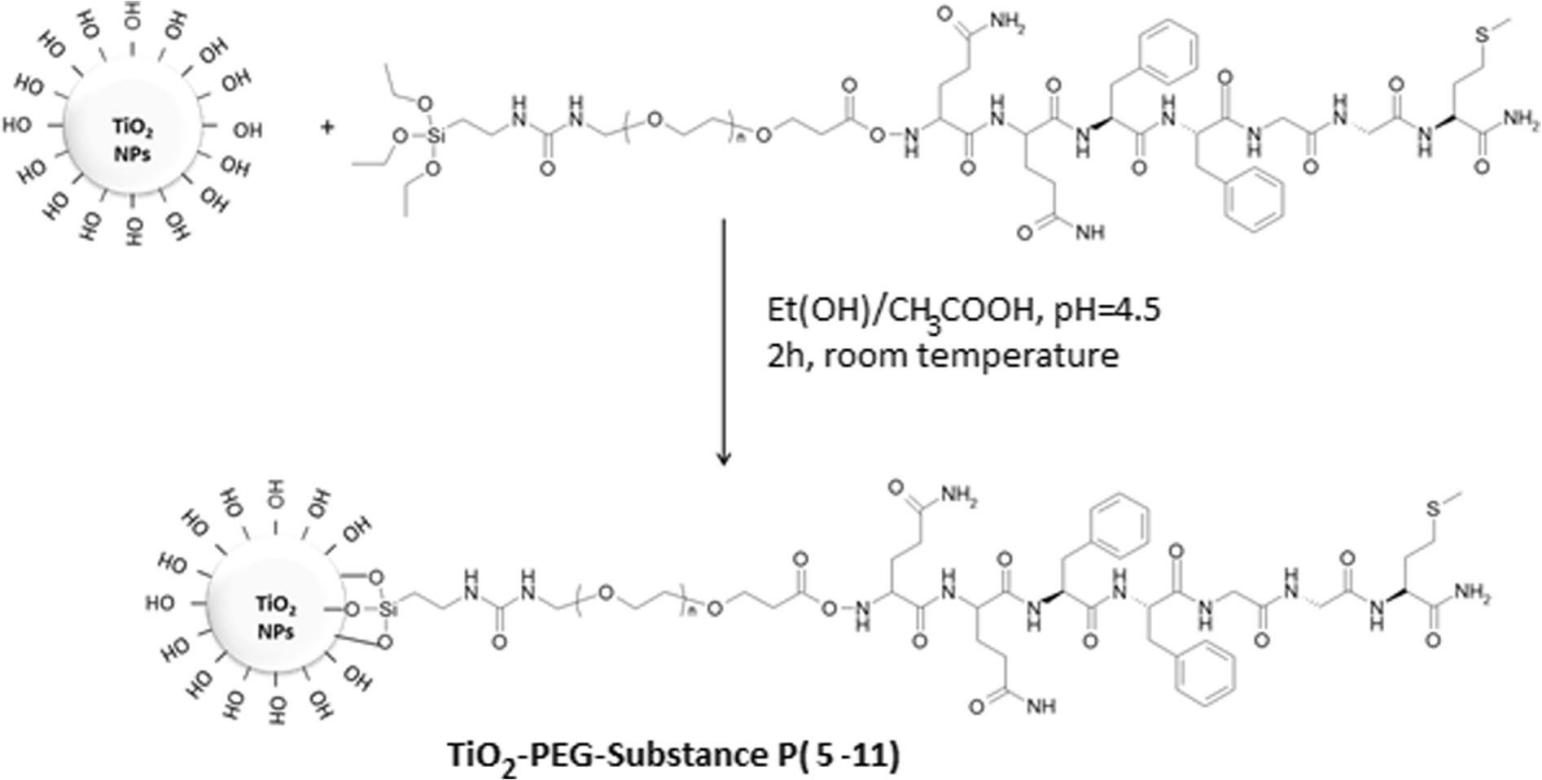
Functionalization of titanium dioxide nanoparticles with PEG-SP(5-11)

Figure 2 shows the procedure for TiO_2_ NPs surface functionalization. In a first step, the silane-PEG-NHS linker was coupled to the SP(5-11) fragment and the obtained bioconjugate silane-PEG-SP(5-11) was attached to the surface of TiO_2_ NPs by siloxane bond formation. Since the OH groups on TiO_2_ surface are active sites for both ^225^Ac adsorption and also for conjugation through silanization process, the number of silane-PEG-SP(5-11) molecules must be low enough to remain sufficient number of OH groups for adsorption of ^225^Ac and its decay products.

**Fig. 3:**
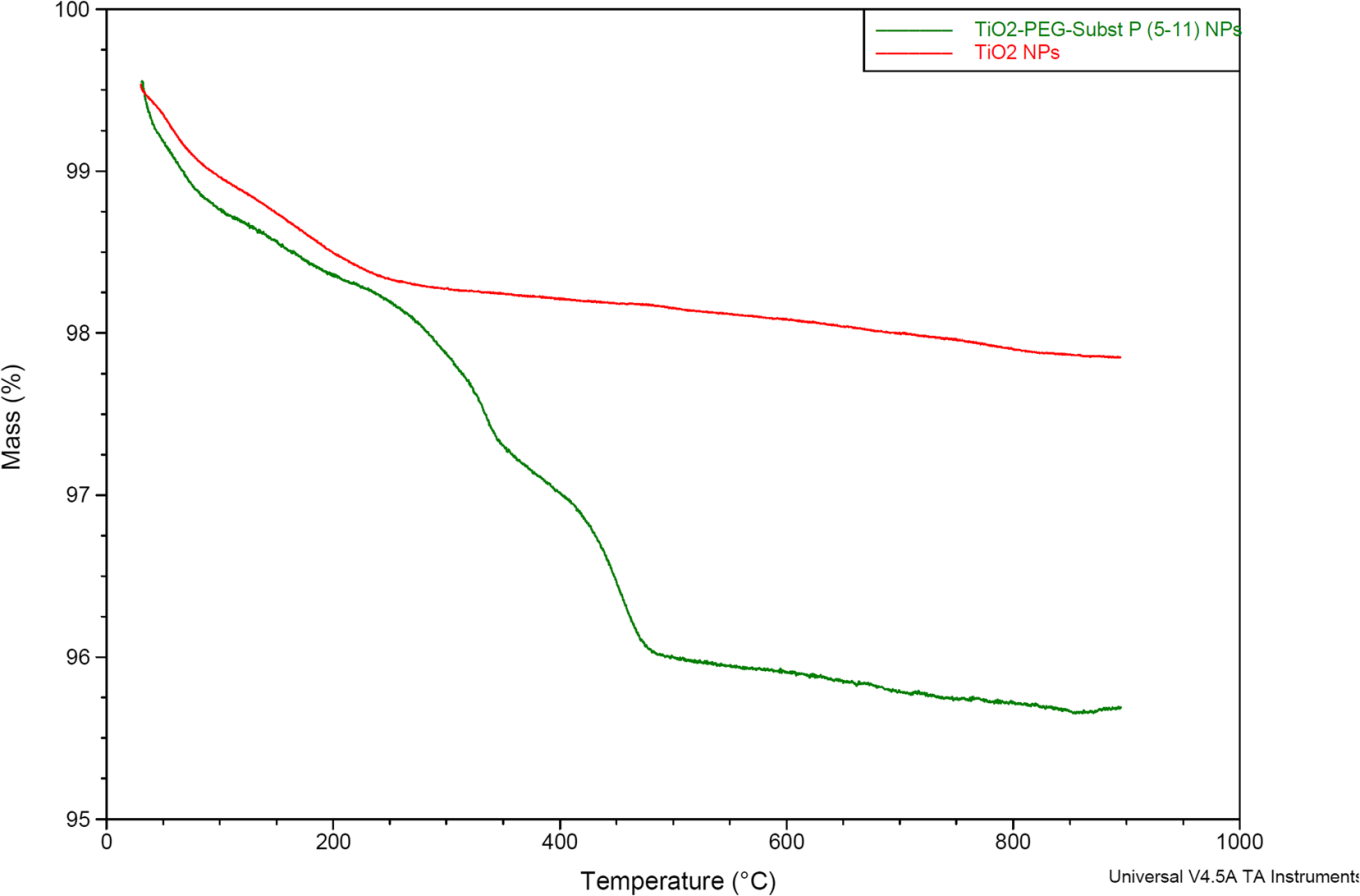
Thermograms of bare TiO_2_ NPs and TiO_2_ NPs functionalized with silane-PEG-SP(5-11)

TGA provided experimental evidence for the presence of silane-PEG-SP(5-11) on the TiO_2_ NPs surface. Figure 3 shows comparison of TGA thermograms of bare TiO_2_ and TiO_2_ functionalized with silane-PEG-SP(5-11). Both thermograms indicate a loss of mass ~ 1% in the range 25–100 °C and additional 0.8% in the range 100–300 °C, which can be attributed to desorption of physically adsorbed and intercrystaline water, respectively. The two additional mass losses can be noticed on the thermogram of functionalized TiO_2_. The first mass loss observed between 270 and 400 °C (1.5%) is due to peptide molecule degradation and the second between 375 and 450 °C (1%) is related to the silane bond degradation.

On the basis of obtained TGA results the number of silane-PEG-SP(5-11) coating molecules per one TiO_2_ nanoparticle can be estimated. The calculation was performed under assumption that the nanoparticle is spherical with medium diameter of 20 nm, as measured by TEM, and that the density of material is 3.8 g cm^−3^. The obtained results show that in average 80 silane-PEG-SP(5-11) molecules are bound to the surface of one TiO_2_ nanoparticle. Taking into account that the ion-exchange capacity of TiO_2_ nanoparticles is about 1 meq g^−1^ (Filipowicz et al. 2014), the number of available OH groups on one TiO_2_ nanoparticle should exceed 3.8 × 10^4^. This indicates that even after functionalization of the TiO_2_ nanoparticle surface with silane-PEG-SP(5-11) molecules, still the great majority of hydroxyl groups remain capable for binding of ^225^Ac^3+^ and formed in the decay process ^221^Fr^+^ and ^213^Bi^3+^ cations.

Bare TiO_2_ NPs and TiO_2_-silane-PEG-SP(5-11) were also characterized by dynamic light scattering (DLS). The obtained values of hydrodynamic diameters and zeta (*ζ*) potentials are presented in Table 1. Due to the hydration layer formed on bare nanoparticles, the hydrodynamic diameter measured by DLS is usually greater than that measured by TEM. The observed increase of the hydrodynamic diameter for TiO_2_ NPs after functionalization process is another confirmation that silanization reaction proceeded and silane-PEG-SP(5-11) molecules were indeed coupled to the surface of TiO_2_ NPs. The measured negative zeta potential value of − 28.5 mV for TiO_2_-silane-PEG-SP(5-11) indicates that particles repel each other and do not aggregate what was confirmed by monitoring changes in hydrodynamic diameter over many days.

**Table 1:** Hydrodynamic diameter and zeta (*ζ*) potential of bare and functionalized TiO_2_ NPs (pH = 7.4, PB buffer)

Nanoparticles	Hydrodynamic diameter	Zeta (*ζ*) potential (mV)
TiO_2_	76.02 ± 5.54	− 23.2
TiO_2_-silane-PEG-SP(5-11)	98.78 ± 4.50	− 28.5

Bare TiO_2_ and TiO_2_-silane-PEG-SP(5-11) NPs were radiolabelled with ^225^Ac (100 kBq) at pH = 6.0 with a high yield of 99.8 ± 2.1% (*n* = 13) and 98.2 ± 1.3% (*n* = 10), respectively. The obtained results indicate that there is no difference in labelling yield between both nanoparticles and that surface functionalization did not have any negative impact on the radiolabelling process. It was expected, as previous calculations confirmed that the number of attached silane-PEG-SP(5-11) molecules is significantly much lower than the number of available OH functional groups responsible for ion-exchange approach with ^225^Ac. However, during our studies, it was found that due to low acidic character of the OH functional groups, the radiolabelling process is pH dependent and in acidic environment (pH = 3) the yield decreases even to only 27% for bare TiO_2_ NPs. Therefore, the optimal pH for radiolabelling reaction is 6.0 and it was used in further studies.

The stability of TiO_2_-silane-PEG-SP(5-11) NPs labelled with ^225^Ac was examined in 0.02 M PBS (pH = 7.4), physiological salt (0.9% NaCl) and cerebrospinal fluid (CSF) for 4 days (Table 2). The leaching of mother radionuclide ^225^Ac and its first decay product ^221^Fr was measured on γ-spectrometer. In PBS and physiological salt NPs retained more than 95% of ^225^Ac and ^221^Fr over 10 days. In CSF, the fraction of ^225^Ac released from NPs was still less than 5%, but the retention of ^221^Fr was decreasing for the first 2 days and later stabilized and remained constant up to 4 days. The high stability in CSF is very important, as treatment of glioblastoma multiforme brain tumour is mostly done through intratumoral or intracavitary injection of radiopharmaceutical, which than can readily penetrate brain parenchyma and target widely disseminated cancerous cells.

**Table 2:** Retention of ^225^Ac and ^221^Fr on TiO_2_-silane-PEG-SP(5-11) NPs radiolabelled with ^225^Ac

Solution	^221^Fr retention (%)			
	24 h	48 h	72 h	96 h
PBS (pH = 7.4)	97.7	98.9	96.8	98.8
Physiological salt (0.9% NaCl)	98.5	98.4	93.3	94.8
Cerebrospinal fluid (CSF)	78.2	70.6	68.5	–

The obtained retention results might be interpreted on the base of recoil energy in the ^225^Ac → ^221^Fr reaction and subsequent *α* decays. Calculated, using the SRIM program (SRIM 2010), recoil range of ^221^Fr in water exceeds 84 nm, but in the material with density close to TiO_2_-anatase shows only about 44 nm. When the decay series is taken into consideration, we can assume that the radionuclides ^217^At, ^213^Bi and ^209^Pb (daughters of ^221^Fr) receive similar recoil energy. The escape of the radioactive isotopes from recoiled daughter and targeting agents allows them to freely migrate in the body, causing toxicity to healthy tissues and decreasing the therapeutic dose delivered to the diseased site. The renal toxicity induced by ^213^Bi is considered to be the major constraint to apply ^225^Ac in a large number of clinical trials (Jaggi et al. 2005; Schwartz et al. 2011). Based on the literature data, one would expect that higher amount of ^221^Fr and ^213^Bi nuclides would be ejected from a 20-nm-diameter TiO_2_ nanoparticle than observed in our studies. The observed smaller release of the daughter radionuclide ^221^Fr (< 5% in PBS and saline solution) suggests that a fraction of the recoil energy takes part in translation of the whole NP rather than being entirely dissipated through atomic displacements resulting, as it occurs in the bulk, in shorter recoil range. Additionally, in the case of TiO_2_ which is an effective cation exchanger, we observe subsequent reloading of escaped recoils ^221^Fr^+^ and ^213^Bi^3+^ as it contains hydroxyl surface groups with high affinity for these cations (Filipowicz et al. 2014; Bogdan et al. 2017; Perekhozheva et al. 1985; Bilewicz et al. 1991). This process can also explain slightly lower stability of radiolabelled NPs in CSF, as in this medium hydroxyl surface groups of NPs are blocked by the proteins and peptides present in CSF what prevents rebinding of released daughter radionuclides.

**Fig. 4:**
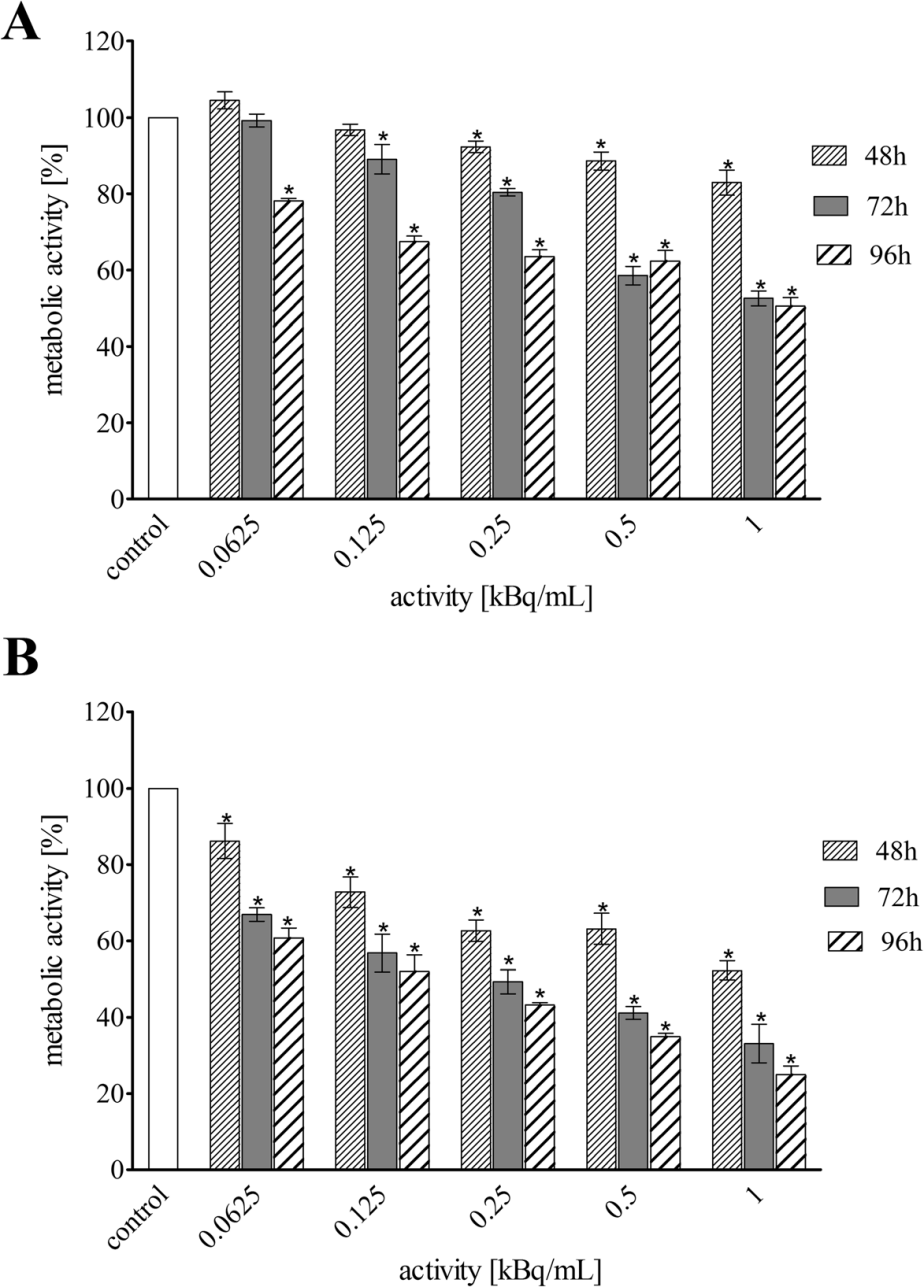
Metabolic activity (MTT assay) of T98G cells treated with different radioactivities of ^**225**^Ac-TiO_2_-silane-PEG (**a**) and ^**225**^Ac-TiO_2_-silane-PEG-SP(5-11) (**b**) NPs for 48, 72 and 96 h. Data are expressed as percent of control and the mean ± SD from three independent experiments

**Fig. 5:**
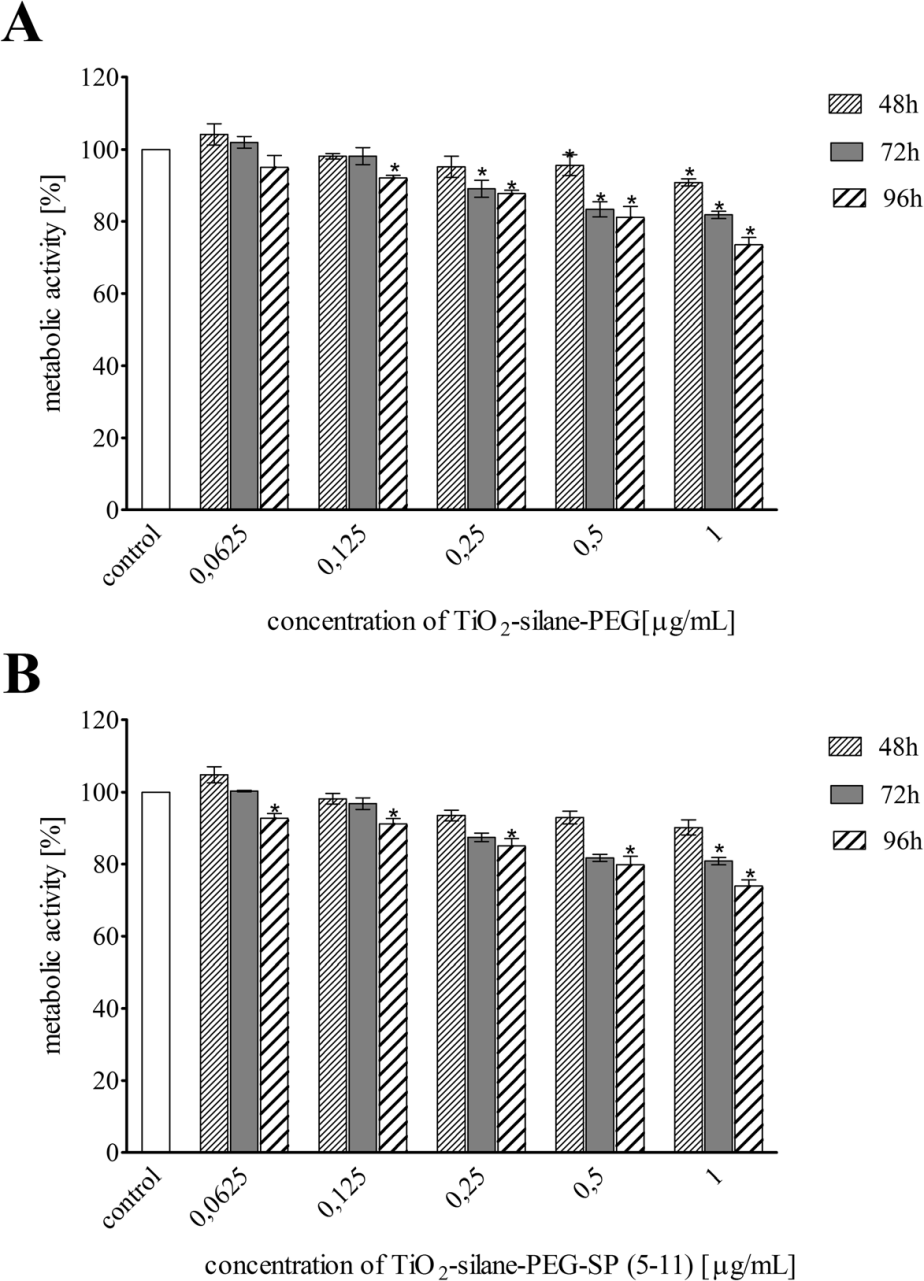
Metabolic activity (MTT assay) of T98G cells treated with different concentration of non-radiolabelled TiO_2_ (**a**) and TiO_2_-silane-PEG-SP(5-11) (**b**) NPs for 48, 72 and 96 h. Data are expressed as percent of control and the mean ± SD from three independent experiments

Cytotoxicity studies were performed on human glioma T98G cells with NK1 receptor overexpression. Cells were exposed to different radioactivities of ^225^Ac-silane-TiO_2_-PEG and ^225^Ac-silane-TiO_2_-PEG-SP(5-11) as well as different concentrations of non-radioactive TiO_2_-silane-PEG and TiO_2_-silane-PEG-SP(5-11) for up to 96 h. The effect of all studied compounds was assessed by the MTT assay. The metabolic activity of cells exposed to ^225^Ac-silane-TiO_2_-PEG-SP(5-11) decreased rapidly with increasing radioactive doses and incubation time (Fig. 4b). On the contrary, ^225^Ac-silane-TiO_2_-PEG NPs control, without targeting biomolecule, reduced cells viability, although the degree was not so much significant. Thus, the obtained data clearly indicate that observed cytotoxicity was specific to NK1 receptors and directly correlated with its expression level on tumour cells. The non-radiolabelled TiO_2_-silane-PEG and TiO_2_-silane-PEG-SP(5-11) NPs were relatively non-toxic as decrease in metabolic activity was observed only for the highest concentration (1 mg/mL) (Fig. 5). This concentration reduced the metabolic activity of T98G cells after 96 h incubation to 75 and 73% of control value for TiO_2_-silane-PEG and TiO_2_-silane-PEG-SP(5-11) NPs, respectively. These results reveal that indeed ^225^Ac attached to NPs is a source of toxicity.

The cytotoxicity of ^225^Ac-silane-TiO_2_-PEG-SP(5-11) is very high in comparison to other radiopharmaceuticals including conjugates radiolabelled with α-emitters like ^213^Bi or ^211^At (Gadbois et al. 1996; Dziawer et al. 2017), however, it is comparable with other radiobioconjugates of ^223^Ra and ^225^Ac (Borchardt et al. 2003; Piotrowska et al. 2017). The decay processes of ^225^Ac and ^223^Ra include four *α* and two *β*^−^ emissions to a stable ^209^Bi and ^207^Pb daughters. Because a very large amount of energy (~ 28 MeV) is released during these decay processes, much smaller amount of radionuclides activity is required to produce the desired effect and ^225^Ac and ^223^Ra radionuclides demonstrate extreme cytotoxicity. Comparison of obtained cytotoxicity results of ^225^Ac-silane-TiO_2_-PEG-SP(5–11) NPs with literature data for radiopharmaceuticals labelled with ^213^Bi (Friesen et al. 2007) demonstrate that several logs less ^225^Ac radioactivity is required to reach similar LD_50_, value, because of the longer half-life and multiple *α*-emissions.

## Conclusion

We have shown that TiO_2_ NPs functionalized with substance P (5-11) can be used to deliver and retain ^225^Ac and its daughter radioisotopes at a target site; thereby reducing the absorbed dose to non-targeted organs. The TiO_2_ NPs successfully retain a large fraction of the ^225^Ac decay products without compromising the tumoricidal properties of the *α* radiation. Because of the reloading of the ^225^Ac decay products the retention efficiencies of TiO_2_ NPs are comparable to the radiolabelled LnPO_4_-Au core shell nanostructures; however, the synthesis process is much simpler and less time-consuming. Furthermore, it has been shown that ^225^Ac-TiO_2_-silane-PEG-SP(5-11) exhibits satisfactory stability in CSF what is very important as it is intended for direct intratumoral or post-resection application. The intravenous injection of the ^225^Ac-TiO_2_-silane-PEG-SP(5-11) is excluded due to the relatively large size and high hydrophilicity which prevents crossing the blood-brain barrier.
